# Effects of ethnicity on the quality of family planning services in Lima, Peru

**DOI:** 10.1186/1472-6963-14-S2-P95

**Published:** 2014-07-07

**Authors:** Maria Elena Planas, Patricia García, Monserrat Bustelo, Cesar Cárcamo, Sebastian Martinez, Hugo Nopo, Julio Rodriguez, Maria-Fernanda Merino, Andrew Morrison

**Affiliations:** 1 School of Public Health and Administration, Universidad Peruana Cayetano Heredia, Lima, Peru; 2 Department of Public Health and Primary Care, Leiden University Medical Centre, Leiden, The Netherlands; 3 Inter-American Development Bank, Washington DC, USA

## Background

Most studies reporting ethnic disparities in the quality of healthcare come from developed countries and rely on observational methods. We conducted the first experimental study to evaluate whether health providers in Peru provide differential quality of care for Family Planning (FP) services, based on the ethnic profile of the patient.

## Materials and methods

In a crossover randomized controlled trial conducted in 2012, a sample of 351 out of the 408 public health establishments in Metropolitan Lima, Peru were randomly assigned to receive unannounced simulated patients enacting indigenous and mestizo (mixed ethnoracial ancestry) profiles (sequence-1) or mestizo and then indigenous profiles (sequence-2), with a five week wash-out period. Both ethnic profiles used the same scripted scenario for seeking contraceptive advice but had distinctive cultural attributes such as clothing, styling of hair, make-up, accessories, posture and patterns of movement and speech. Our primary outcome measure of quality of care is the proportion of technical tasks performed by providers, as established by Peruvian FP clinical guidelines. Providers and data analysts were kept blinded to the allocation. The trial was registered with ClinicalTrials.gov NCT01885858.

## Results

We found a non-significant mean difference of -0.7% (p=0.23) between ethnic profiles in the percentage of technical tasks performed by providers. However we report large deficiencies in compliance with the quality standards of care for both profiles.

## Conclusions

Differential provider behaviour based on the patient’s ethnic profiles compared in the study did not contribute to deficiencies in FP outcomes observed. The study highlights the need to explore other determinants for poor compliance with quality standards, including demand and supply side factors, and calls for interventions to improve the quality of care for FP services in Metropolitan Lima.

